# Implementation of a digital tool for population management in Primary Health Care

**DOI:** 10.11606/s1518-8787.2023057005321

**Published:** 2024-04-01

**Authors:** Debora Paulino da Silva Almeida, Paulo Leandro de Oliveira, Glauber Alves dos Prazeres, Lorrayne Belotti, Jessica Domingues, Natalia Martins Bonassi, Ilana Eshriqui, Renata Soares Martins, Leticia Yamawaka de Almeida, Daiana Bonfim

**Affiliations:** I Hospital Israelita Albert Einstein Albert Einstein Center for Studies, Research, and Practices in Primary Health Care and Networks Sao Paulo Brazil Hospital Israelita Albert Einstein - Albert Einstein Center for Studies, Research, and Practices in Primary Health Care and Networks, Sao Paulo, Brazil

**Keywords:** Territorialization in Primary Care, Community Health Workers, Primary Health Care, Electronic Health Records, Population Health Management

## Abstract

**OBJECTIVE:**

Describe the implementation of a digital diagnostic and territorial monitoring tool in primary healthcare.

**METHODS:**

Quantitative and qualitative study, developed in 14 basic healthcare units in São Paulo, with community health workers, coordinators, nurses, and physicians. Data collection occurred in four phases: analysis of the instruments used by the team for territory management; development of the digital tool; training and implementation; and evaluation after 90 days using focus groups. Descriptive analyses were conducted by calculating absolute and relative frequencies to treat quantitative data. Qualitative data were subjected to content analysis.

**RESULTS:**

Three hundred thirty-four professionals participated in the study. In the first step, territory management’s main challenges were filling out various instruments, system failures, data inconsistency, internet infrastructure/network, and lack of time. Therefore, a digital tool was developed consisting of 1) a spreadsheet recording the number of family members and markers of health conditions, date of visit, and number of return visits; 2) a spreadsheet with a summary of families visited, not visited, and refusals; and 3) a panel with a summary of the data generated instantly. In the evaluation, after the initial use of the tool, the themes that emerged were integration of the tool into daily work, evaluation of the digital tool implementation process, and improvement and opportunities for improvement.

**CONCLUSIONS:**

Faced with the challenges faced by family healthcare teams when filling out systems and managing the territory, the tool developed provided greater reliability and agility in data visualization, reduced the volume of instruments, and optimized the work process.

## INTRODUCTION

While the organization of healthcare services in the Health Care Network (HCN) has been discussed in Brazil since 2011^1^, there are many challenges related to the incorporation of this process into professional practice, caused, among other factors, by the lack of integration of healthcare systems, a reality present in different contexts^2^.

One of the proposals for the HCN to be operationalized refers to breaking management based on supply to incorporate the population base, which comprises broad knowledge of the territory, including factors related to the social determinants of health (SDH) and the epidemiological, environmental, and cultural status^3^.

However, due to the adoption of care models for acute conditions, supply management, and fragmentation of the healthcare system, this is still a challenge to be overcome. In this context, Health Care Planning (HCP) emerges as a methodology used to organize the HCN, with one of its main fronts being the strengthening and organization of Primary Health Care (PHC) work processes^3,4^.

From this perspective, territorialization is configured as a basic macro-process of PHC, essential for the population-based management of the HCN, comprising registration and classification of families’ vulnerabilities, local diagnosis, identification, and stratification of target subpopulations by risk factor or health conditions^5^.

However, a challenge in the healthcare system, especially in developing countries, is promptly obtaining and making reliable data and information available for decision-making. In Brazil, recording data that generate health information is the responsibility of the entire Family Healthcare Strategy (FHS), with the Community Health Workers (CHW) playing a fundamental role in this process, as it is, in most cases, the main link between the team and families, in addition to being responsible for registering these users in the service^6^.

In this context, in 2013, the e-SUS Primary Health Care (e-SUS PHC) was established in PHC, a national health information system (NHIS) that aims to meet different local computerization needs, optimizing the process data collection and support for care coordination.

However, despite the notable progress in the implementation of the NHIS, there is still a concern with the reliability of the information extracted for decision-making^7,8^, as well as a weakness regarding the imputation, monitoring, and presentation of data practically for use in teams. Furthermore, most reports are generated in a consolidated format, making it difficult to plan and target actions for specific populations.

Thus, understanding the need to equip CHWs with technological tools that support data and information for the population-based management of the family healthcare team, this study aimed to describe the implementation of a digital diagnostic and territorial monitoring tool in the PHC.

## METHODS

### Study Design

This is a quantitative-qualitative study developed in two steps. In phase 1 of quantitative data collection, the combination of approaches made diagnosing the context related to the CHW work process possible. The qualitative approach, conducted in phase 4, allowed the evaluation of the tool in use from the perspective of the CHWs involved in the implementation process.

### Study Site

The study, developed between August and December 2022, was conducted in 14 Basic Healthcare Units (BHU) in the Campo Limpo and Vila Andrade administrative districts, South Zone of São Paulo, which have around 400,198 inhabitants. Currently, BHUs have a total of 92 FHS, 30 oral healthcare teams, six Multidisciplinary Primary Care Teams (MPCT), the Green and Healthy Environments Program (GHEP), and 298,616 people registered in e-SUS PHC, of which 86% have no healthcare insurance.

In 2019, the implementation of the HCP methodology began in these services, intending to organize processes in PHC, including territorialization, allowing the qualification of care based on an understanding of population-based management.

### Population

Seven hundred ten professionals were invited to participate in the study, including 108 physicians, 115 nurses, 459 CHWs, 14 senior nurses, and 14 coordinators. The inclusion criteria for participation were working as a physician, nurse, CHWs, senior nurse, or BHU coordinator. The exclusion criteria were being on vacation or away from work during the data collection period.

### Data Collection and Analysis

#### Phase 1: Analysis of the instruments used by the team for territory management

A diagnostic questionnaire composed of open and closed questions was developed in Microsoft Forms, covering aspects such as sociodemographic characteristics, length of experience in the healthcare sector and the territory, tools used for territory management, access, and forms of use of data in user care management.

Subsequently, the questionnaire was applied to CHWs, nurses, physicians, and coordinators. The resulting data was stored in a Microsoft Office Excel spreadsheet. Descriptive quantitative data analyses were carried out by calculating absolute and relative frequencies. Content analysis was done by categorizing the answers to the open questions in the questionnaire^9^.

#### Phase 2: Tool development

In the context of implementing the digital tool entitled FAMILY (Material Suplementar)^a^, the pre-existing infrastructure of tablets, which had already been made available for incorporation into the CHWs routine, was considered. These devices have a 4G connection, provided by the São Paulo Municipal Health Department (SPMHD), which ensures the readiness of essential technological resources to carry out their tasks.

FAMILY was inspired by the paper-only version offered by SPMHD, known by the teams as hive or schedule, corresponding to a checkered sheet with the family numbers in ascending order. In this, the professional can include the date of the home visit and the health conditions of that family, identified by symbols defined by the service. The CHWs fills out a new form at the beginning of the following month, identifying all health conditions again.

Its development was organized in three steps, carried out by the team of analysts and specialists from the Innovation and Digital Health Center: 1) analysis of the operationalization process of the hive printed in the CHW visit and monitoring routine, which aims to obtain clarification about its use to record health markers/conditions of families and monitored users; 2) structuring the fields to be filled in and defining the markers to be inserted into the digital tool, namely: systemic arterial hypertension, diabetes mellitus, bedridden patients, children under 1-year-old, children aged 1 to 2 years old, pregnant women, leprosy, tuberculosis, syphilis in pregnant women, congenital syphilis, and alert; and 3) development of the tool using Microsoft Office 365 package Excel®, applying formulas and conditional formatting.

During the development of the tool, a key consideration was ensuring the security and privacy of patient data, as well as accessibility to healthcare professionals, especially in areas where internet infrastructure can be a challenge. As the tool is a spreadsheet on the Office 365 Excel platform, it offers robust security and access control features that ensure compliance with the General Data Protection Law. Access requires authorization provided by the unit manager and logging in with an institutional email address on the network, guaranteeing data traceability.

Furthermore, each CHWs has restricted access only to its spreadsheet, in which data is recorded by family, not by individual, and presented in a consolidated and non-nominal format. The only identifier in family information is its number, preventing direct access to individual data. To obtain information about a specific individual, such as their health conditions, the CHWs or any other healthcare professional must access official systems, such as *e-SUS Territory*, where they can locate the family number and then identify the individual.

It is essential to highlight that this process is traceable and controlled, requiring personal and non-transferable logins and passwords. In addition, information security training is carried out, all professionals sign confidentiality agreements, and the technology and information security management team constantly monitors data processing on the network.

FAMILY evaluation and approval were conducted through monitored tests in a production environment on the tablet of five CHWs who received training. Notably, at this step, the need for adjustments to step 3 of FAMILY was identified for the other teams.

#### Phase 3: Training and implementation

**Figure 1:**
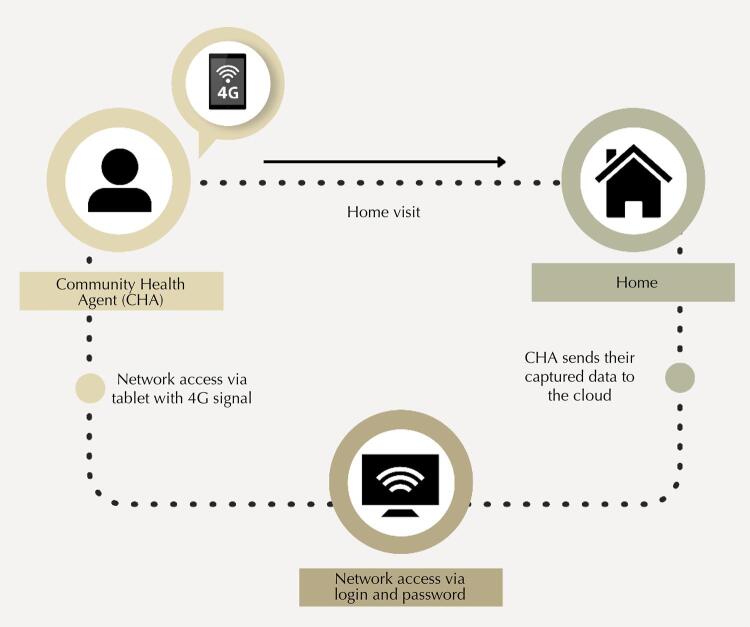
Flow of information collected by the community health worker. Tools and criteria used by nurses, physicians, and coordinators for territory management and home visits.

Face-to-face training was carried out to implement FAMILY at the 14 BHUs, lasting one hour each, with the participation of 387 CHWs and representatives of the multidisciplinary team. On these occasions, all the functionalities of the new tool and the flow of information were presented (Figure 1).

During the implementation of the instrument, professionals were able to present suggestions for new markers to be included to meet local needs, and these suggestions were taken to discussions with the institutional committees responsible for the lines of care so that there could be analysis and conceptualization of each marker to be inserted when updating new versions of the tool.

After implementation in the 14 BHUs, the development team monitored data completion fortnightly, sent reports to correct the necessary information by professionals, and visited each unit to clarify doubts after using the tool.

#### Phase 4: Evaluation of tool implementation

Two focus groups were gathered in one hour of remote meetings to understand the CHW’ perception of the process of implementing and using the tool after 90 days of use. In total, 28 CHWs participated, representing the 14 BHU. Two professionals per unit were invited, indicated by the coordinator based on the criteria: one CHW with easiness and one CHW who had difficulty using FAMILY, aiming to guarantee the heterogeneity of the groups.

The data were transcribed, and two researchers conducted the content analysis according to the steps described by Graneheim and Lundman^10^. The coding was carried out independently by the researchers, considering the manifest content of the textual data. The codes were categorized based on similarities between words and text fragments. Subsequently, they were organized into three themes, considering the latent content of the units of analysis. In the end, with the support of a third researcher, the inconsistencies were discussed and standardized.

## Ethical Aspects

The present study was approved by the Ethics Committee of Albert Einstein Israelite Hospital (approval IEC/IRB 3.674.106, CAAE 12395919.0.0000.0071) and by the Research Ethics Committee of the São Paulo Municipal Health Department (approval IEC/IRB 3.617.970, CAAE 12395919.0.3001.0086).

## RESULTS

Three hundred thirty-four professionals participated in the analysis step of the instruments used by the team to manage the territory (step 1). Of these, 280 were CHW, 6 unit coordinators, 27 nurses, and 16 physicians. Most participants were female at birth (92.8%) and self-reported brown race/skin color (52.7%). Among higher education professionals, white race/color was predominant (51.9%). Approximately 20% of CHWs had completed higher education or were studying it (Table 1).

**Table 1:** Descriptive analysis of participants.

Variables	Community health workers		Other professionals		Total	
		
	n	%	n	%	n	%
Sex at birth^a^						
Female	264	94.6	45	83.3	309	92.8
Male	15	5.4	9	16.7	24	7.2
Total	279	100	54	100	333	100
Race/color^a^						
Yellow	1	0.4	5	9.3	6	1.8
Caucasian	77	27.7	28	51.9	105	31.6
Brown	157	56.5	18	33.3	175	52.7
Black	43	15.5	3	5.6	46	13.9
Total	278	100	54	100	332	100
Education level^a^						
Complete primary education	20	7.2	0	0	20	6
Complete high school	199	71.3	0	0	199	59.9
Graduated	31	11.1	5	10	36	10.8
Studying higher education	24	8.6	0	0	24	7.2
Specialization	3	1.1	34	68	37	11.1
Master’s degree and/or doctorate	2	0.7	11	22	13	3.9
Total	279	100	50	100	329	99.1
Time at PHC						
Less than one year	40	14.3	3	5.6	43	12.9
Between 1 and 3 years	82	29.3	6	11.1	88	26.4
Between 4 and 8 years	87	31.1	19	35.2	106	31.8
More than nine years	71	25.4	26	48.1	97	29.1
Total	280	100	54	100	334	100.3
Time in current position						
Less than one year	43	15.4	8	14.8	51	15.3
Between 1 and 3 years	80	28.6	18	33.3	98	29.3
Between 4 and 8 years	87	31.1	16	29.6	103	30.8
More than nine years	70	25	12	22.2	82	24.6
Total	280	100	54	100	334	100
Time in the territory						
Less than one year	40	14.3	14	25.9	54	16.2
Between 1 and 3 years	82	29.3	19	35.2	101	30.2
Between 4 and 8 years	87	31.1	14	25.9	101	30.2
More than nine years	71	25.4	7	13	78	23.4
Total	280	100	54	100	334	100

PHC: Primary Health Care.

^a^Variables that do not include the total sample, given that the response is not mandatory when collecting data.

When asked about using tools to collect and manage territory information, most CHWs reported using notebooks to record information (42.6%) and define cases to be discussed with the team (54.5%). The majority also stated that they use at least two tools to map and identify pregnant women (45.7%), chronic patients (50.9%), and children under two years old (51.4%) living in the territory. Furthermore, 44.1% did not update the data daily, and 46.4% reported that the address was the main criterion for defining the visits to be carried out, with the production form (33.6%) for the CHWs being the main form of monitoring families that had not yet received visits (Table 2).

**Table 2:** Tools and criteria used by community health workers for territory management.

Variables	Community health workers	

	n	%
Tool used to record information^a^		
Notebooks	232	42.6
Printed hive	91	16.7
Spreadsheets	125	23
Others	96	17.6
Total	544	100
Easiness for updating territory data^b^		
No	69	25
Yes	207	75
Total	276	100
Reason for not updating data^a^		
Lack of time	11	13.6
I have no registered data	4	4.9
Data loss	56	69.1
Others	10	12.3
Total	81	100
Data update frequency^b^		
Daily	152	55.9
Weekly	51	18.8
Fortnightly	10	3.7
Monthly	53	19.5
Every two months	5	1.8
Every six months	1	0.4
Total	272	100
Main criterion for defining the visits that will be carried out^b^		
Randomly	31	11.2
By priority groups (pregnant women, children, chronic patients)	82	29.7
By streets and addresses	128	46.4
By team request	5	1.8
By user request	5	1.8
By vulnerability	8	2.9
Others	17	6.2
Total	276	100
Method to monitor families that have not yet received a visit in the month^a^		
Notebooks	81	16.9
Printed hive	81	16.9
E-SUS	62	12.9
Production form	161	33.6
By memory	36	7.5
Not monitored	3	0.6
Others	55	11.5
Total	479	100
Method to record cases to be discussed with the team^a^		
Notebooks	216	54.5
Printed hive	9	2.3
E-SUS	19	4.8
Production form	62	15.7
By memory	48	12.1
Others	42	10.6
Total	396	100

^a^Questions with more than one possible answer.

^b^Variables that do not include the total sample, given that the response is not mandatory when collecting data.

Among the other professionals, 66.7% reported not managing the agenda based on the territory’s characteristics. Despite this, 51.9% and 57.9% reported accessing the territory’s data in an updated and easy manner, respectively. The tools and methods indicated as the primary means of accessing territory data were spreadsheets (18.4%), contact with CHW (17.5%), and team meetings (17.1%). Most used one or two tools to identify pregnant women, chronic patients, and children under two years of age in the territory (Table 3).

**Table 3:** Tools and criteria used by nurses, physicians, and coordinators for territory management.

Variables	Other professionals	

	n	%
Carrying out agenda management based on the characteristics of the territory		
No	36	66.7
Yes	18	33.3
Total	54	100
Access to your territory’s data in an up-to-date manner		
No	26	48.1
Yes	28	51.9
Total	54	100
Access to your territory’s data in an easy manner		
No	23	42.6
Yes	31	57.4
Total	54	100
Tool(s)/method(s) used to access your territory’s data^a^		
Printed hive	7	3.1
Contact with CHW	40	17.5
Spreadsheets	42	18.4
PEC	33	14.5
Power BI	25	11
Team meeting	39	17.1
SIGA-Saúde	35	15.4
Others	7	3.1
Total	228	100
Frequency of data access		
Daily	12	22.2
Weekly	17	31.5
Fortnightly	2	3.7
Monthly	18	33.3
Every two months	4	7.4
Every six months	1	1.9
Total	54	100
Territory scenarios where updates can be monitored in real-time^a^		
Children under two years old	16	7.1
Chronic patients	32	14.2
Pregnant women	39	17.3
Leprosy patients	14	6.2
Tuberculosis patients	31	13.7
Registered population	17	7.5
Congenital syphilis and in pregnant women	30	13.3
Total registered families	22	9.7
Total families visited	15	6.6
I do not follow up on any of the above	10	4.4
Total	226	100

PEC: Electronic Citizen Medical Record; CHW: Community Health Worker; SIGA Saúde: Integrated Health Care Management System

^a^Questions with more than one possible answer

^b^- Variables that do not include the total sample, given that the response is not mandatory when collecting data.

Among the territory management tools self-reported by the team of coordinators, physicians, and nurses, the following stood out: spreadsheets (n = 26), meetings (n = 10), consultation with the CHW (n = 7), indicator portal (n = 7), reports (n = 9), registration and forms (n = 5), home visit (n = 6), and chat application (n = 3). Furthermore, the main challenges for accessing the territory’s data were several systems (n = 7), system failure (n = 4), data inconsistency (n = 5), internet infrastructure/network (n = 12), and lack of time (n = 6).

Regarding the reasons for not monitoring the territory’s data in real-time, the main ones identified were lack of time (n = 12), difficulty with internet/infrastructure (n = 5), lack of standardization (n = 4), and difficulty with the tool (n = 4). When the CHWs were asked about the criteria used to take a case for discussion with the team, vulnerability (n = 65), need (n = 58), urgency (n = 42), patient demand (n = 36), complexity (n = 32), priority (n = 30), and subpopulations (n = 28) were the main themes raised by professionals.

During the FAMILY implementation process, suggestions for adjustments by FHS professionals were presented and inserted, such as total home visits pending in the month and “CHW panel: status of home visits.”

Furthermore, in a BHU, it was necessary to include two other pieces of information in the “menu” tab: “no information on the occupation of the property located in a condominium that restricts access to the CHW” and “no registration due to lack of access to residents of properties in condominiums that restricts CHW entry.” Thus, the team can identify the area with registration potential and plan their actions, considering that this territory is mainly composed of buildings and has particularities regarding the territorialization process, access to condominiums, and distribution of the number of families.

**Figure 2:**
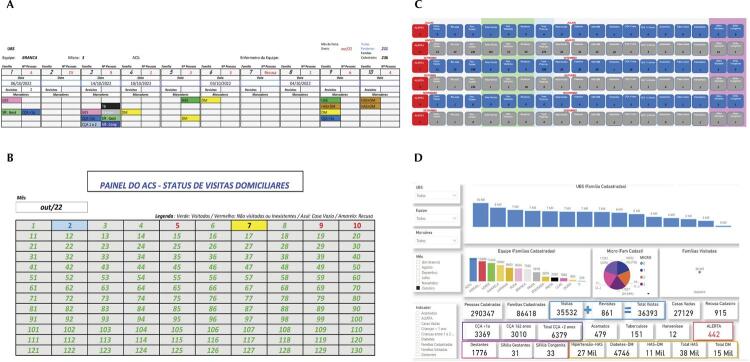
FAMILY tool and Power BI. São Paulo, 2022

Note: 2a: Spreadsheet recording the number of family members and markers; 2b: Spreadsheet with a summary of families visited, not visited, and refusals; 2c: Panel with a summary of data generated on time; 2d: Power BI with indicators from all FHS.

Figure 2 presents the final implemented tool, and Power BI. FAMILY comprises a spreadsheet recording the number of family members and the possibility of health condition markers, date of visit and number of return visits, a summary of families visited, not visited, and refusals. Furthermore, the panel with data summary is generated instantly for immediate use by the CHW and the team.

After using FAMILY, the CHWs evaluated the integration of the tool into daily work based on the description of its use in routine activities and the perceived repercussions on the organization of the work process and productivity. They also presented some elements related to acceptability, challenges, and barriers to routine use. Furthermore, based on their experience, they made suggestions for improvement and opportunities for improvement in the tool, especially concerning reviewing existing elements and including new fields (Chart 1).

**Chart 1:** Themes, categories, codes, and their evidence from content analysis. São Paulo, 2022.

Theme	Category	Code	Evidence/fragments	
Integration of the tool into daily work	Repercussions on the organization of the work process and productivity	Data reliability	“(...) what it measures is much more reliable, you know, because it brings us a much more real number” (GF2)	“(...) it is great, it brings very real numbers” (GF2)
		Agility in data visualization	“I think it’s a cool [tool]. I think it’s exactly that, you know, everything in the area is more visible” (GF1)	“I agree with what she said. There is also that alert part, you know, and we can fill in that he has diabetes and is taking medication (...) it is easier for us to see, locating this view helped a lot, it helped a lot, it helped rally a lot ” (GF1)
		Reduction in the volume of instruments used and optimization of time	“(...) so I don’t need to take all those files to know who the hypertensive people are and separate them there, there is a map of my area” (GF2)	“ (...) the fact that you have the entire work process at hand there during the visit. I do the visit at e-SUS and launch it at the hive is very quick, you know, and saves time” (GF1)
	Usability in routine activities	Territory characterization/mapping and subpopulation identification	“(...) the hive really helps a lot, you know, in characterizing the territory, it really helps, you know. As we already mentioned, it helps us to know, yeah, how much of the population, how many children there are who are less than one year old, less than two years old, how many children, how many... yes, older people, hypertensive people, these things that are on the marker today (...) I can identify through the hive where are the empty houses, where are the houses with people that I have to offer registration, where are the houses that have already refused registration” (GF2)	“(...) we can do a study of the area, you know, this area has more hypertensive people, you know, this area has more elderly people” (GF2)
		Planning and monitoring home visits	“it also helps a lot with the visits we conduct to know exactly when it happened so as not to repeat it (...) In the hive, we can know exactly what day it was done so we don’t have to do it again, we have all this control of return visits, this part is also very good” (GF1)	“We see our visits daily; we have greater control of the visits we missed and then the amount that has already been conducted” (GF2)
		Production record	“In the closing, we use now everything based on in the hive (...) I use it to know how many pregnant women there are (...) I think that in everyday life we use the hive data a lot, a lot, really a lot. In everyday life, we use it a lot. There is no day that I don’t open my hive or to visit, or to consult some data, there isn’t a day” (GF2)	“In closing, we used a lot to have a real base of our territory” (GF2)
Assessment of the digital tool implementation process	Acceptability of the digital tool	Satisfaction, usability, and relevance	“(...) to measure indicators like this, it’s great, I loved it, which is actually our schedule, but digitalized, you know, so I particularly liked it a lot, I just have to complement it. I wouldn’t change anything, just increase, just improve, so to speak” (GF2)	“for example, my question is always this bureaucratic issue of papers because if you have a device with all this potential to be explored and then you are part of the digital work, we stay in the unit and have to deal with the paper, so the hive is much better precisely because of this because I’m in the patient’s home, I can conduct the visit at ESUS and fill in the hive because everything is right there at hand” (GF1)
		Availability for change, adaptation, and professional engagement	“It was fascinating because as everything new creates a certain concern, the bad thing is when it was said that the hive would be implemented, we were kind of like, wow, more work is coming. As my colleague reported, sometimes we have too much demand, you know, and then it is a big concern that demand will increase. However, when we started doing it, on the contrary, it will be very positive, as has already been said, it helps a lot, at least in closing. At the end of the month, it’s exciting” (GF1)	“I still had difficulty in the first month, you know, because it really wasn’t just about the hive. There were other things too that we needed to deal with over time, but now this month I’ve felt that for me, concerning the hive, we already have a different reality, more as a help, indeed (...)” (GF1)
Assessment of the digital tool implementation process	Challenges and barriers to routine use	Technological resource restrictions	“(...) it doesn’t make much sense for you to have access because sometimes it takes a long time depending on where you are, you know, at least my territory is made up of alleys, so I have difficulty accessing in this territory, you know, And so, it sometimes takes a while to open, you know” (GF2)	“(...) I think that when there are a lot of people using it at the same time, it has to sort it out, you know? I have to update it, so I close the tab and open it again” (GF1)
		Preference for other resources/strategies	“The easiness of having a lot of time in the area is that I have a good part of all this in my head, you know. The hive is more for closing; it’s not so common in my daily life” (GF2)	
		Complex use of the digital tool	“(...) several colleagues here, who from experience, you know, we see, and we help, have a lot of difficulties in return visits and placing return visits in the hive” (GF2)	“Recently, we had a new CHW on our team, and she was really lost seeing the hive. She didn’t understand much (...) it was very novel for us, we had little training time, you know, to be able to learn and insert it. (...) training one day, and the next, I was on vacation. When I came back, I was racking my brain so that I couldn’t explain myself, so it was a little difficult” (GF2)
		Dynamics of the home visit	“We have the report in our hand, we have the tablet in our hand, and then we have to put it in the hive, and whether we like it or not, it delays the visit a little, you know” (GF2)	“I try to make and use my hive at the actual time of my visit. Sometimes, it’s a little complicated, considering there’s so much we have to do with the visit, which is conducting it on the tablet, plus doing the report, filling out the hive, answering the patient’s doubts, and passing on instructions to the patient. So, sometimes we start to eliminate some things that are not essential for us to do there to optimize the time in visit further, you know (...)” (GF2)
Improvement and opportunities for improvement	Review of existing elements	Adaptation in markers	“And yes, there is a lot to improve too. (...) because there are hypertensive and diabetic patients combined (...) I think it could also be separated, you know” (GF2)	“(...) maybe put some field because there is a refusal from CHW and a refusal from the patient who doesn’t want us to go, so there are two types of refusal. I know that there is one in the field, you know, but I think it could also add something about it because there isn’t, you know” (GF2)
		Layout adjustments	“(...) there’s one thing that bothers me: when I enter the hive, I find the space very small, you know? The space we have to be able to put it there and the dates I think are poor, concerning when you are going to fill it out. Since we are talking about improvements, I think the visualization of the instrument is not as efficient for me. Sometimes, I put the number there, for example, but then the other part of the screen disappears, so I can’t see it. I have to wait a while for this part to return to insert these numbers on the rest, so I think it has to improve in this area.” (GF1)	
	Inclusion of new fields	Inclusion of report field	“(...) and the question that doesn’t want to mention that it would be even more wonderful if it could also have a report in it or in another tool like the one from EPHEALTH.” (GF1)	“I think 99% of CHWs agree that if you could include the report in the hive, it would be wonderful, perfect” (GF1)
		Inclusion of observation field	“(...) if there was a field to fill in information that both the nurse and the physician could view, I think it would be much more effective” (GF2)	
		Inclusion of new subpopulation markers	“(...) I think that we have a lot more than just hypertensive people, diabetics, pregnant women, those who are bedridden, you know, those who we follow up, there are older people, you know, that we follow up, there are women of childbearing age. These are points that we also need to take into account, you know, we need to have control” (GF2)	“Or what the colleague pointed out, about the markers that are in our goal were also included in the hive (...) so if we could put all the markers that we had to follow, it would be quite interesting” (GF1)
		Inclusion of nominal identification of subpopulations	“It would be interesting to highlight who are the hypertensive and diabetic people” (GF2)	“I think trying to mark, having some marker so that another person can visualize who that person is” (GF2)
		Inclusion of field quantifying families and visits conducted	“Do you know what I think is missing from the hive? (...) the number of visits on that day, got it?” (GF2)	“(...) one thing I also have a little difficulty with, which I keep seeing, is to total the number of families, let’s assume: at this moment I now have 213 families just so I can have this count, I have to keep counting them one by one, you know?” (GF2)

e-SUS: Strategy that refers to the qualified computerization process of the Unified Health System (SUS) in search of an electronic SUS (e-SUS); EPHEALTH: Private software company for Community Health Workers; CHW: Community Health Worker.

GF1: Focus group 1; GF2: Focus group 2.

## DISCUSSION

The main instruments used in territory management and for access to data by the eSF were spreadsheets and notebooks by the CHWs, resulting in difficulties in managing this information. The biggest challenges reported for accessing the territory’s data were internet infrastructure/network, multiple information systems for recording information, and lack of time. After the initial use of FAMILY, from the perspective of the CHWs, essential aspects were raised to enhance the tool in the daily practice of teams in PHC, such as the integration of the tool in the daily work, the evaluation of the implementation process of the digital tool, the improvement and opportunities for improvement.

The work of the CHW, integrated with the multidisciplinary team in the FHS, is fundamental for understanding the territory based on the process of territorialization in healthcare. However, daily practice highlights weaknesses in the work process and communication with the rest of the team, aggravated by disconnected information systems, as demonstrated by national studies^2,11^.

Most CHWs reported difficulty updating territory information due to data loss after feeding the systems. The findings converge with results from research conducted in Kenya and Malawi, which investigated the coherence of data reported by community health workers in the context of community health programs in low- and middle-income countries. Only 15% of the data collected by the community health workers were found to be consistently reported to their supervisors^12^. This congruence of results points to recurring challenges that affect data quality in different contexts: the unavailability of adequate tools for collecting and reporting information, deficiencies in the training and supervision of CHWs, and the absence of effective quality control mechanisms and inadequate records.

Participants in this study reported that they needed to access approximately six tools to identify data from three target subpopulations. This trend of fragmentation persists in Brazil and is a challenge to be overcome so that population-based management becomes a reality in PHC. From 2013 to 2018, 31 national health information systems were available in use in PHC, and, among them, 15 did not have any unification of interfaces with PEC/e-SUS PHC^2^.

Mitigation of the problems identified in the pre-implementation phase of the tool was addressed in the World Health Organization (WHO) Guidelines)^13^ on health policy and systemic support for optimizing CHW programs. In this sense, FAMILY presents itself as a tool with the potential to contribute to the organization and optimization of data collection and monitoring in the PHC territory, promoting greater security and reliability.

When we evaluated the “integration of the tool into daily work,” the tool proved to help in carrying out territorial diagnosis, as well as in monitoring and organizing the eSF work process, meeting the assumptions established by HCP regarding the macro-process of territorialization and the need for knowledge of the enrolled population, including social and health aspects, characterization and registration of subpopulations, alerts and registration of these individuals and the need to implement effective NHIS^5^.

Adopting mobile technologies in CHW work can improve the work process, develop assertive interventions, and more complete, high-quality, and timely data collection. A systematic review of North American literature showed that, compared to paper use, the use of technologies allowed reduced errors and data loss, greater ease in real-time review and analysis for decision-making, and a quick response to health problems^14^.

Another challenge to be overcome is the incorporation of using territory information in decision-making and organization of the work process in the daily life of PHC units, an essential action in the context of HCP. Although more than half of professionals report having access to data, only a few reported structuring their agendas based on this information. From this perspective, the implementation of FAMILY, which is easy to use in the CHW’s work routine, integrated with other healthcare service professionals, enhances teamwork, communication, recognition of cases that need greater attention, the prioritization of care and population-based management and the findings demonstrated in the analysis of the codes present in the CHWs’ statements related to usability, favoring the organization of PHC macro and micro processes, through the HCP.

Regarding the process of implementing the digital tool, although there is recognition of its usability and relevance, there are reports by CHWs of the limits of using the technology related to handling and understanding, difficulty in accessing the internet in some locations, fear of using the tablet due to the risk of theft, and temporary slowness in accessing the tool.

In a qualitative study with higher education professionals who worked in Family Health teams in Ceará, despite the teams hardly using information and communication technologies (ICT), they recognized that their use would provide greater speed in accessing information and ease in evaluation of care interventions provided. They also identified the need for adequate recording and archiving of information, which using ICT^15^ can facilitate.

The 2030 Agenda for Sustainable Development recognizes the need to increase access to communication and information technologies. Mobile wireless technologies for public health, or mHealth, are an integral part of e-Health, which refers to the economic and secure use of information, communication, and technologies to support healthcare. In this sense, digital technologies are becoming an essential resource for providing healthcare services^16^.

Despite being central to developing healthcare systems, implementing digital health technologies can be challenging in low- and middle-income countries. The WHO recognizes that it is essential to invest in efforts to overcome the main impediments developing countries face in using new digital health technologies, such as enabling environment, infrastructure, education, human and financial capacity, internet connectivity, and technology ownership. Given this situation, collaboration between member countries is proposed, with the creation of national policies designed for the reality of each country, guiding the development of strategies^17^.

Regarding the improvement and opportunities for improving FAMILY, highlights were the CHW’ desire to include a field for recording the visit report to reduce further the number of working instruments, including the nominal list of people and new markers. Since adjustments can be made throughout the implementation process, FAMILY’s acceptability, applicability, and power as a support tool for population-based management is demonstrated.

Despite the satisfactory results and the possibility of adjusting the tool to different realities, the limitations of this study involve the specific scenario in which the research was carried out. Developing new studies that analyze the challenges and particularities of implementing FAMILY in different regions is essential. Furthermore, it should be noted that the tool does not operate offline, meaning its functionality depends on a constant 4G connection. In cases where the 4G connection is unavailable, the CHW makes the records in physical paper format and, later, enters the information into the tool when connectivity is restored. However, it is a low-cost development tool, and adaptations can be made according to the profile and needs of the territory.

## CONCLUSION

In conclusion, with the implementation of FAMILY, there is greater reliability and agility in data visualization, a reduction in the volume of manual instruments, and optimization of time, allowing a visual mapping of the territory through the use of colors and spatial organization of families in a same panel, thus transforming data into information that is easily accessible to CHW and FHS. It should also be noted that the tool does not replace any existing information system in PHC but offers additional and complementary functionalities, such as vulnerability mapping, alerts for prioritizing care, facilitated access to reports and performance indicators, and an intuitive interface for real-time data visualization.

It is expected that FAMILY, available in the supplementary material, can be incorporated by other municipalities to contribute to operationalizing basic PHC macro processes and improve population-based management, as proposed by HCP.

## Footnotes

a Material Suplementar

## References

[1] Ministério da Saúde (BR) (2010). Portaria nº 4.279/2010. Estabelece diretrizes para a organização da Rede de Atenção à Saúde no âmbito do Sistema Único de Saúde (SUS). Diário Oficial União.

[2] Coelho FC, Andreazza R, Chioro A (2021). Integração entre os sistemas nacionais de informação em saúde: o caso do e-SUS Atenção Básica. Rev Saude Publica.

[3] Evangelista MJO, Guimarães AMDN, Dourado EMR, Vale FLB, Lins MZS, Matos MAB (2022). O planejamento e a construção das Redes de Atenção à Saúde no DF, Brasil. Cienc Saude Colet.

[4] Mendes EV, Matos MAB, Evangelista MJO, Barra RP (2019). A construção social da atenção primária à saúde.

[5] Ministério da Saúde (BR), Sociedade Beneficente Israelita Brasileira Albert Einstein, Instituto Israelita de Responsabilidade Social (2019). PLANIFICASUS: guia Workshop 2 território e gestão de base populacional.

[6] Bueno AS, Calliari ABT, Emmanouilidis J, Braz MA, Moura FRR, Brew MC (2019). Mapeamento georreferenciado de doenças crônicas em unidade de atenção primária de Porto Alegre. Rev Saude Cien.

[7] Cielo AC, Raiol T, Silva EN, Barreto JOM (2022). Implantação da estratégia e-SUS Atenção Básica: uma análise fundamentada em dados oficiais. Rev Saude Publica.

[8] Pinheiro ALS, Andrade KTS, Silva DO, Zacharias FCM, Gomide MFS, Pinto IC (2016). Health management: the use of information systems and knowledge sharing for the decision making process. Texto Contexto - Enferm.

[9] Bardin L (2011). Análise de conteúdo.

[10] Graneheim UH, Lundman B (2004). Qualitative content analysis in nursing research: Concepts, procedures and measures to achieve trustworthiness. Nurse Educ Today.

[11] Pedebos LA, Rocha DK, Tomasi Y (2018). A vigilância do território na atenção primária: contribuição do agente comunitário na continuidade do cuidado. Saúde Debate.

[12] Regeru RN, Chikaphupha K, Kumar MB, Otiso L, Taegtmeyer M (2020). 'Do you trust those data?': a mixed-methods study assessing the quality of data reported by community health workers in Kenya and Malawi. Health Policy Plan.

[13] World Health Organization, Organisation for Economic Co-Operation and Development, The World Bank (2018). Delivering quality health services: a global imperative for universal health coverage.

[14] Braun R, Catalani C, Wimbush J, Israelski D (2013). Community health workers and mobile technology: a systematic review of the literature. PLoS One.

[15] Mota DN, Torres RAM, Guimarães JMX, Marinho MNASB, Araújo AF (2018). Tecnologias da informação e comunicação: influências no trabalho da estratégia Saúde da Família. J Health Inform.

[16] World Health Organization (2018). mHealth: use of appropriate digital technologies for public health: report by the Director-General In: Seventy-first world health assembly.

[17] World Health Organization (2021). Global strategy on digital health 2020-2025.

