# Supplementary material for: Implementation of a digital tool for population management in Primary Health Care
Source: Rev Saude Publica. 2024 Apr 1;57(Suppl 3):6s. doi: 10.11606/s1518-8787.2023057005321 (PMC11037907; doi:10.11606/s1518-8787.2023057005321)
Supplement: Translation

# Translation: Implementação de ferramenta digital para gestão populacional na atenção primária à saúde

**Authors:** Debora Paulino da Silva Almeida, Paulo Leandro de Oliveira, Glauber Alves dos Prazeres, Lorrayne Belotti, Jessica Domingues, Natalia Martins Bonassi, Ilana Eshriqui, Renata Soares Martins, Leticia Yamawaka de Almeida, Daiana Bonfim

**Affiliations:** I Brasil Hospital Israelita Albert Einstein. Centro de Estudos, Pesquisa e Prática em Atenção Primária e Rede. São Paulo, SP, Brasil

**Keywords:** Territorialização da Atenção Primária, Agentes Comunitários De Saúde, Atenção Primária à Saúde, Registro Eletrônico de Saúde, Gestão da Saúde da População

## Abstract

**OBJETIVO:**

Descrever a implementação de uma ferramenta digital de diagnóstico e monitoramento territorial na atenção primária à saúde.

**MÉTODOS:**

Estudo quanti-qualitativo, desenvolvido em 14 Unidades Básicas de Saúde do município de São Paulo, com agentes comunitários de saúde, coordenadores, enfermeiros e médicos. A coleta de dados ocorreu em quatro fases: análise dos instrumentos utilizados pela equipe para gestão do território; desenvolvimento da ferramenta digital; treinamento e implantação; avaliação após 90 dias por meio de grupos focais. Foram realizadas análises descritivas por meio do cálculo de frequências absolutas e relativas para tratamento dos dados quantitativos. Os dados qualitativos foram tratados pela análise de conteúdo.

**RESULTADOS:**

334 profissionais participaram do estudo. Na primeira etapa foram identificados como principais desafios para gestão do território o preenchimento de diversos instrumentos, falhas no sistema, inconsistência dos dados, infraestrutura/rede de internet e falta de tempo. Assim, foi desenvolvida uma ferramenta digital composta por: i) planilha com registro do número de membros familiares e marcadores de condições de saúde, data da visita e quantidade de revisitas; ii) planilha com resumo de famílias visitadas, não visitadas e recusas; e iii) um painel com resumo dos dados gerados instantaneamente. Na avaliação, após uso inicial da ferramenta, as temáticas que emergiram foram: integração da ferramenta no cotidiano de trabalho; avaliação do processo de implementação da ferramenta digital; aperfeiçoamento e oportunidades de melhoria.

**CONCLUSÃO:**

Frente aos desafios encontrados por parte das equipes de saúde da família para preenchimento dos sistemas e gestão do território, a ferramenta desenvolvida proporcionou maior fidedignidade e agilidade na visualização dos dados, redução no volume de instrumentos e otimização do processo de trabalho.

## INTRODUÇÃO

A organização dos serviços de saúde em Rede de Atenção à Saúde (RAS) vem sendo discutida no Brasil desde 2011^1^, contudo, muitos são os desafios relacionados à incorporação desse processo na prática profissional, ocasionados, entre outros fatores, pela falta de integração dos sistemas de saúde, realidade presente em diversos contextos^2^.

Uma das propostas para que a RAS seja operacionalizada refere-se ao rompimento da gestão com base na oferta, para a incorporação da gestão com base populacional, a qual compreende o amplo conhecimento do território, incluindo fatores relacionados aos Determinantes Sociais da Saúde (DSS) e a situação epidemiológica, ambiental e cultural^3^.

Entretanto, em virtude da adoção de modelos de atenção às condições agudas, gestão de ofertas e fragmentação do sistema de saúde, este ainda é um desafio a ser superado. Neste contexto, a Planificação da Atenção à Saúde (PAS) surge como uma metodologia utilizada para organização de RAS, sendo uma de suas frentes principais, o fortalecimento e organização dos processos de trabalho da atenção primária à saúde (APS)^3,4^.

Nesta perspectiva, a territorialização configura-se como um macroprocesso básico da APS, essencial para a gestão de base populacional da RAS, compreendendo cadastramento e classificação de vulnerabilidades das famílias; diagnóstico local; identificação e estratificação das subpopulações alvo por fator de risco ou condições de saúde^5^.

Porém, um desafio presente no sistema de saúde, principalmente de países em desenvolvimento, é a obtenção e disponibilização em tempo oportuno de dados e informações fidedignos para tomada de decisão. No Brasil, o registro de dados, que geram informações de saúde, é uma atribuição de toda a equipe de saúde da família (eSF), e o agente comunitário de saúde (ACS) tem papel fundamental nesse processo, por ser na maioria das vezes o principal elo entre a equipe e as famílias, além de se caracterizar como o responsável pelo cadastro desses usuários no serviço^6^.

Neste contexto, em 2013, foi estabelecida na APS a estratégia e-SUS Atenção Primária (e-SUS APS), um sistema de informação em saúde (SIS) nacional que se propõe a atender as diferentes necessidades locais de informatização, com otimização do processo de coleta de dados e apoio à coordenação do cuidado.

Todavia, apesar do notório avanço na implantação do SIS, ainda se observa uma preocupação com a fidedignidade das informações extraídas para tomada de decisão^7,8^, bem como uma fragilidade no que tange à imputação, monitoramento e apresentação dos dados de forma prática para uso das equipes. Além disso, a maioria dos relatórios são gerados no formato consolidado, fato que dificulta o planejamento e direcionamento de ações para populações específicas.

Assim, entendendo a necessidade de instrumentalizar os ACS com ferramentas tecnológicas que subsidiem dados e informações para a gestão de base populacional da equipe de saúde da família, este estudo teve como objetivo descrever a implementação de uma ferramenta digital de diagnóstico e monitoramento territorial na APS.

## MÉTODOS

### Desenho do Estudo

Trata-se de um estudo quanti-qualitativo, desenvolvido em duas etapas. A combinação das abordagens oportunizou, na fase 1 da coleta de dados, de natureza quantitativa, realizar um diagnóstico do contexto relacionado ao processo de trabalho dos ACS. A abordagem qualitativa, conduzida na fase 4, permitiu a avaliação da ferramenta em uso a partir da perspectiva dos ACS envolvidos no processo da sua implantação.

### Local de Estudo

O estudo, desenvolvido no período entre agosto e dezembro de 2022, foi realizado em 14 unidades básicas de saúde (UBS) localizadas nos distritos administrativos Campo Limpo e Vila Andrade, Zona Sul do município de São Paulo, que contam com cerca de 400.198 habitantes. Atualmente as UBS possuem um total de 92 eSF, 30 equipes de saúde bucal, 06 Núcleos de Apoio à Saúde da Família (NASF), Programa Ambientes Verdes e Saudáveis (PAVS) e 298.616 pessoas cadastradas no e-SUS APS, das quais 86% não possui plano de saúde.

Em 2019, foi iniciada a implantação da metodologia da PAS nestes serviços com o objetivo de organizar processos na APS, entre estes, o de territorialização, permitindo a qualificação do cuidado a partir do entendimento da gestão de base populacional.

### População

Foram convidados 710 profissionais para participarem do estudo, sendo: 108 médicos(as), 115 enfermeiros(as), 459 ACS, 14 enfermeiros(as) sêniores e 14 coordenadores(as). O critério de inclusão para participação foi: atuar como médico, enfermeiro ou ACS de eSF ou atuar como enfermeiro sênior ou coordenador de UBS. Foram considerados como critérios de exclusão: estar de férias ou afastado do cargo durante o período de coleta de dados.

### Coleta e Análise dos Dados

#### Fase 1: análise dos instrumentos utilizados pela equipe para gestão do território

Foi desenvolvido, no *Forms office 365 survey*, um questionário diagnóstico composto por questões abertas e fechadas que contemplavam aspectos como as características sociodemográficas, tempo de atuação na área da saúde e no território, ferramentas utilizadas para gestão do território, acesso aos dados e formas de utilização dos dados na gestão do cuidado dos usuários.

Posteriormente, o questionário foi aplicado para ACS, enfermeiros, médicos e coordenadores. Os dados provenientes foram armazenados no programa Microsoft Office Excel. Análises descritivas dos dados quantitativos foram realizadas por meio do cálculo de frequências absolutas e relativas. Análise de conteúdo foi realizada por meio de categorização das respostas das perguntas abertas presentes no questionário^9^.

#### Fase 2: desenvolvimento da ferramenta

No contexto da implementação da ferramenta digital intitulada FAMILY (Material Suplementar), foi levada em consideração a infraestrutura pré-existente de *tablets*, que já haviam sido disponibilizados para incorporação à rotina dos ACS. Esses dispositivos possuem uma conexão 4G, disponibilizada pela Secretaria Municipal de Saúde de São Paulo (SMSSP), que assegura a prontidão dos recursos tecnológicos essenciais para a execução de suas tarefas.

A FAMILY foi inspirada na versão disponível, somente em papel, ofertada pela SMSSP conhecida pelas equipes como colmeia ou cronograma, a qual corresponde a uma folha quadriculada, com os números das famílias em ordem crescente. Nesta, o profissional pode incluir a data da visita domiciliar e as condições de saúde daquela família, identificadas por símbolos definidos pelo serviço. No início do mês subsequente, o ACS realiza o preenchimento de um novo impresso, identificando todas as condições de saúde novamente.

O seu desenvolvimento foi organizado em três etapas desempenhadas pelo time de analistas e especialista do Núcleo de Inovação e Saúde Digital: 1) análise do processo de operacionalização da colmeia impressa na rotina de visita e monitoramento do ACS, com o intuito de obter esclarecimento acerca de sua utilização para o apontamento dos marcadores/condições de saúde das famílias e usuários acompanhados; 2) estruturação dos campos a serem preenchidos e definição dos marcadores a serem inseridos na ferramenta digital, sendo eles: hipertensão arterial sistêmica; diabetes mellitus; acamados; crianças menores 1 ano; crianças de 1 a 2 anos; gestantes; hanseníase; tuberculose; sífilis em gestante; sífilis congênita; e alerta; 3) desenvolvimento da ferramenta com a utilização do aplicativo Excel® do pacote Office 365 da Microsoft, aplicando fórmulas e formatação condicional.

Durante o desenvolvimento da ferramenta, uma consideração fundamental foi a garantia da segurança e privacidade dos dados dos pacientes, bem como a acessibilidade aos profissionais de saúde, especialmente em áreas onde a infraestrutura de internet pode ser um desafio. Como a ferramenta é uma planilha na plataforma Excel do Office 365, oferece recursos robustos de segurança e controle de acesso que garantem a conformidade com a Lei Geral de Proteção de Dados. Para acessá-la é necessária autorização fornecida pelo gestor da unidade e realização de login na rede, com e-mail institucional, fato que garante a rastreabilidade de dados.

Ainda, cada ACS tem acesso restrito apenas à sua planilha, onde os dados são registrados por família, não por indivíduo, e são apresentados de forma consolidada e não nominal. O único identificador presente nas informações da família é o número dessa família, o que impede o acesso direto a dados individuais. Para obter informações sobre um indivíduo específico, como suas condições de saúde, o ACS ou qualquer outro profissional de saúde deve acessar os sistemas oficiais, como o e-SUS Território, onde podem localizar o número da família e, em seguida, identificar o indivíduo.

É importante destacar que esse processo é rastreável e controlado, exigindo logins e senhas pessoais e intransferíveis. Em complemento são aplicados treinamentos de segurança da informação, assinatura de termos de confidencialidade por todos os profissionais e monitoramento constante dos dados tramitados em toda rede pela equipe de gestão da tecnologia e segurança da informação.

Foram realizadas a avaliação e homologação da FAMILY, por meio de testes monitorados em ambiente de produção no *tablet* de cinco ACS que receberam treinamento. Destaca-se que, nesta fase, foi identificada a necessidade de ajustes para a etapa 3 da FAMILY às demais equipes.

#### Fase 3: treinamento e implantação

Para implantação da FAMILY foram realizados treinamentos presenciais nas 14 UBS, com uma hora de duração, com a participação de 387 ACS e representantes da equipe multiprofissional. Nessa ocasião, foram apresentadas todas as funcionalidades da nova ferramenta e o fluxo de informações (Figura 1).

**Figura 1:** Fluxo da informação coletada pelo agente comunitário de saúde durante as Tabela 3. Ferramentas e critérios utilizados pelos enfermeiros, médicos e coordenadores para gestão do território.visitas domiciliares.

Durante a implantação do instrumento, os profissionais puderam apresentar sugestões de novos marcadores a serem incluídos, a fim de atender às necessidades locais, e estas sugestões foram levadas para discussões com os comitês institucionais responsáveis pelas linhas de cuidado para análise e conceituação de cada marcador a ser inserido na atualização de novas versões da ferramenta.

Após a implantação nas 14 UBS, a equipe de desenvolvimento realizou monitoramento quinzenal do preenchimento dos dados, encaminhou relatórios para correção das informações necessárias pelos profissionais, e realizou uma visita em cada UBS para alinhamento de dúvidas após a utilização da ferramenta.

#### Fase 4: avaliação da implantação da ferramenta

Com o objetivo de conhecer a percepção dos ACS sobre o processo de implantação e uso da ferramenta, após 90 dias de uso foram realizados dois grupos focais, no formato remoto, com duração de uma hora cada. No total, participaram 28 ACS representando as 14 UBS. Foram convidados dois profissionais por unidade, indicados pelo coordenador, a partir dos critérios: um ACS que apresentava facilidade e um ACS que apresentava dificuldade na utilização da FAMILY, visando garantir a heterogeneidade dos grupos.

Os dados foram transcritos e dois pesquisadores realizaram a análise de conteúdo, de acordo com as etapas descritas por Graneheim e Lundman^10^. A codificação foi realizada de forma independente pelos pesquisadores, considerando o conteúdo manifesto dos dados textuais. Os códigos foram agrupados em categorias baseadas em semelhanças entre palavras e fragmentos de texto. Posteriormente, as categorias foram organizadas em três temas considerando o conteúdo latente das unidades de análise. Ao final, com o apoio de um terceiro pesquisador, realizou-se a discussão e uniformização das inconsistências.

## Aspectos Éticos

O presente estudo foi aprovado pelo Comitê de Ética em Pesquisa do Hospital Israelita Albert Einstein (parecer CEP 3.674.106, CAAE 12395919.0.0000.0071) e pelo Comitê de Ética em Pesquisa da Secretaria Municipal de Saúde de São Paulo (parecer 3.617.970, CAAE 12395919.0.3001.0086).

## RESULTADOS

Participaram da etapa de análise dos instrumentos utilizados pela equipe para gestão do território (etapa 1), 334 profissionais. Destes, 280 eram ACS, seis coordenadores(as) de unidade, 27 enfermeiros(as) e 16 médicos(as). A maioria dos participantes era do sexo de nascimento feminino (92,8%) e autorreferida raça/cor parda (52,7%). Entre os profissionais de nível superior, a raça/cor branca foi predominante (51,9%). Aproximadamente 20% dos ACS tinham ensino superior completo ou estavam cursando (Tabela 1).

**Tabela 1:** Análise descritiva dos participantes do estudo.

Variáveis	Agente comunitário de saúde		Outros profissionais		Total	
		
	n	%	n	%	n	%
Sexo de nascimento^a^						
Feminino	264	94,6	45	83,3	309	92,8
Masculino	15	5,4	9	16,7	24	7,2
Total	279	100	54	100	333	100
Raça/cor^b^						
Amarela	1	0,4	5	9,3	6	1,8
Branca	77	27,7	28	51,9	105	31,6
Parda	157	56,5	18	33,3	175	52,7
Preta	43	15,5	3	5,6	46	13,9
Total	278	100	54	100	332	100
Nível de escolaridade^a^						
Ensino fundamental completo	20	7,2	0	0	20	6
Ensino médio completo	199	71,3	0	0	199	59,9
Superior completo	31	11,1	5	10	36	10,8
Superior cursando	24	8,6	0	0	24	7,2
Especialização	3	1,1	34	68	37	11,1
Mestrado e/ou doutorado	2	0,7	11	22	13	3,9
Total	279	100	50	100	329	99,1
Tempo na APS						
Menos de 1 ano	40	14,3	3	5,6	43	12,9
Entre 1 e 3 anos	82	29,3	6	11,1	88	26,4
Entre 4 e 8 anos	87	31,1	19	35,2	106	31,8
Há mais de 9 anos	71	25,4	26	48,1	97	29,1
Total	280	100	54	100	334	100,3
Tempo no cargo atual						
Menos de 1 ano	43	15,4	8	14,8	51	15,3
Entre 1 e 3 anos	80	28,6	18	33,3	98	29,3
Entre 4 e 8 anos	87	31,1	16	29,6	103	30,8
Há mais de 9 anos	70	25	12	22,2	82	24,6
Total	280	100	54	100	334	100
Tempo no território						
Menos de 1 ano	40	14,3	14	25,9	54	16,2
Entre 1 e 3 anos	82	29,3	19	35,2	101	30,2
Entre 4 e 8 anos	87	31,1	14	25,9	101	30,2
Há mais de 9 anos	71	25,4	7	13	78	23,4
Total	280	100	54	100	334	100

APS: atenção primária à saúde.

^a^Variáveis que não contemplam a amostra total, tendo em vista a não obrigatoriedade da resposta na coleta dos dados.

Quando questionados sobre o uso de ferramentas para coleta e gerenciamento das informações do território, grande parte dos ACS referiu que utilizava cadernos para registros das informações (42,6%) e definição dos casos a serem discutidos com a equipe (54,5%). A maioria referiu também que fazia uso de pelo menos duas ferramentas para realizar o mapeamento e identificação de gestantes (45,7%), crônicos (50,9%) e crianças menores de 2 anos (51,4%) residentes no território de atuação. Além disso, 44,1% não atualizavam os dados diariamente e 46,4% relataram que o endereço era o principal critério para definição das visitas a serem realizadas, sendo para os ACS, a principal forma de monitoramento das famílias que ainda não receberam visitas, a ficha de produção (33,6%) (Tabela 2).

**Tabela 2:** Ferramentas e critérios utilizados pelos agentes comunitários de saúde para gestão do território.

Variáveis	Agente comunitário de saúde	

	n	%
Ferramenta utilizada para registro das informações^a^		
Cadernos	232	42,6
Colmeia impressa	91	16,7
Planilhas	125	23
Outros	96	17,6
Total	544	100
Facilidade para atualização dos dados do território^b^		
Não	69	25
Sim	207	75
Total	276	100
Motivo para não atualização dos dados^a^		
Falta de tempo	11	13,6
Não tenho dados registrados	4	4,9
Perda de dados	56	69,1
Outros	10	12,3
Total	81	100
Periodicidade de atualização dos dados^b^		
Diariamente	152	55,9
Semanalmente	51	18,8
Quinzenalmente	10	3,7
Mensalmente	53	19,5
A cada 2 meses	5	1,8
A cada 6 meses	1	0,4
Total	272	100
Principal critério para definição das visitas que serão realizadas^b^		
De forma aleatória	31	11,2
Por grupos prioritários (gestantes, crianças, crônicos)	82	29,7
Por ruas e endereços	128	46,4
Por solicitação da equipe	5	1,8
Por solicitação do usuário	5	1,8
Por vulnerabilidade	8	2,9
Outros	17	6,2
Total	276	100
Forma de monitoramento das famílias que ainda não receberam visita no mês^a^		
Cadernos	81	16,9
Colmeia impressa	81	16,9
E-SUS	62	12,9
Ficha de produção	161	33,6
Pela memória	36	7,5
Não monitora	3	0,6
Outros	55	11,5
Total	479	100
Forma de registro dos casos a serem discutidos com a equipe^a^		
Cadernos	216	54,5
Colmeia impressa	9	2,3
E-SUS	19	4,8
Ficha de produção	62	15,7
Pela memória	48	12,1
Outros	42	10,6
Total	396	100

^a^Questões com mais de uma possibilidade de resposta.

^b^Variáveis que não contemplam a amostra total, tendo em vista a não obrigatoriedade da resposta na coleta dos dados.

Dentre os demais profissionais, 66,7% reportaram que não realizavam gestão da agenda com base nas características do território. Apesar disso, 51,9% e 57,9% relataram ter acesso aos dados do território de forma atualizada e facilitada, respectivamente. As ferramentas e métodos indicados como principais meios para acessar dados do território foram: planilhas (18,4%); contato com ACS (17,5%); e, reunião de equipe (17,1%). A maioria utilizava uma ou duas ferramentas para identificar gestantes, crônicos e crianças menores de 2 anos no território (Tabela 3).

**Tabela 3:** Ferramentas e critérios utilizados pelos enfermeiros, médicos e coordenadores para gestão do território.

Variáveis	Outros profissionais	

	n	%
Realização da gestão da agenda com base nas características do território		
Não	36	66,7
Sim	18	33,3
Total	54	100
Acesso aos dados do seu território de forma atualizada		
Não	26	48,1
Sim	28	51,9
Total	54	100
Acesso ao dado do seu território de forma facilitada		
Não	23	42,6
Sim	31	57,4
Total	54	100
Ferramenta(s)/método(s) utilizados para acessar os dados do seu território^a^		
Colmeia impressa	7	3,1
Contato com ACS	40	17,5
Planilhas	42	18,4
PEC	33	14,5
Power BI	25	11
Reunião de equipe	39	17,1
SIGA Saúde	35	15,4
Outros	7	3,1
Total	228	100
Periodicidade de acesso aos dados		
Diariamente	12	22,2
Semanalmente	17	31,5
Quinzenalmente	2	3,7
Mensalmente	18	33,3
A cada 2 meses	4	7,4
A cada 6 meses	1	1,9
Total	54	100
Cenários do território em que consegue acompanhar as atualizações em tempo real^a^		
Crianças menores de 2 anos	16	7,1
Crônicos	32	14,2
Gestantes	39	17,3
Pacientes com hanseníase	14	6,2
Pacientes com tuberculose	31	13,7
População cadastrada	17	7,5
Sífilis congênita e em gestantes	30	13,3
Total de famílias cadastradas	22	9,7
Total de famílias visitadas	15	6,6
Não acompanho nenhuma das anteriores	10	4,4
Total	226	100

^a^ Questões com mais de uma possibilidade de resposta

^b^ Variáveis que não contemplam a amostra total, tendo em vista a não obrigatoriedade da resposta na coleta dos dados.

Entre as ferramentas de gestão do território autorreferidas pela equipe de coordenadores, médicos e enfermeiros, destacaram-se: planilhas (n = 26), reuniões (n = 10), consulta ao ACS (n = 7), portal de indicadores (n = 7), relatórios (n = 9), cadastro e fichas (n = 5), visita domiciliar (n = 6), whatsapp (n = 3). E os principais desafios para acesso aos dados do território foram: diversos sistemas (n = 7), falha no sistema (n = 4), inconsistência dos dados (n = 5), infraestrutura/rede de internet (n = 12), falta de tempo (n = 6).

Com relação aos motivos do não acompanhamento dos dados do território em tempo real, identificou-se, principalmente, a falta de tempo (n = 12), dificuldade com internet/infraestrutura (n = 5), falta de padronização dos dados (n = 4), dificuldade com a ferramenta (n = 4). Quando os ACS foram questionados sobre os critérios utilizados para levar um caso para discussão com a equipe, observou-se que vulnerabilidade (n = 65), necessidade (n = 58), urgência (n = 42), demanda do paciente (n = 36), complexidade (n = 32), prioridade (n = 30) e subpopulações (n = 28) foram os principais temas levantados pelos profissionais.

No processo de implantação da FAMILY sugestões de adequações por parte dos profissionais da eSF foram apresentadas e inseridas, tais como: total de visitas domiciliares pendentes no mês e “painel do ACS: status de visitas domiciliares”.

Além disso, em uma UBS foi necessário incluir duas outras informações na aba “menu”: “sem informação sobre a ocupação do imóvel localizado em condomínio que restringe acesso ao ACS” e “sem cadastro por falta de acesso aos moradores de imóveis em condomínios que restringe a entrada do ACS”. Desta forma, é possível a equipe identificar sua área com potencial de cadastro e planejar suas ações, considerando que esse território é composto principalmente por prédios, e possui particularidades com relação ao processo de territorialização, acesso aos condomínios e distribuição do número de famílias.

A ferramenta final implantada e o Power BI são apresentados na Figura 2. A FAMILY foi composta por: planilha com registro do número de membros familiares e possibilidade de marcadores de condições de saúde, data da visita e quantidade de revisitas, resumo de famílias visitadas, não visitadas e recusas. Além disso, o painel com resumo dos dados é gerado de forma instantânea para uso imediato do ACS e da equipe.

**Figura 2:** Ferramenta FAMILY e Power BI. São Paulo, 2022

Nota: 2a: Planilha com registro do número de membros familiares e marcadores; 2b: Planilha com resumo de famílias visitadas, não visitadas e recusas; 2c: Painel com resumo dos dados gerados *on time*; 2d: Power BI com indicadores de todas as eSF.

Após o uso da FAMILY, os ACS avaliaram a integração da ferramenta no cotidiano de trabalho a partir da descrição do seu uso em atividades de rotina e das repercussões percebidas na organização do processo de trabalho e produtividade, apresentaram alguns elementos relacionados a aceitabilidade bem como desafios e barreiras para uso na rotina. Além disso, a partir da experiência, realizaram sugestões para o aperfeiçoamento e oportunidades de melhoria na ferramenta, especialmente ao que se refere à revisão de elementos existentes e a inclusão de novos campos (Quadro).

**Quadro:** Temas, categorias, códigos e suas evidências da análise de conteúdo. São Paulo, 2022.

Tema	Categoria	Código	Evidência/fragmentos	
Integração da ferramenta no cotidiano de trabalho	Repercussões na organização do processo de trabalho e produtividade	Fidedignidade dos dados	“(...) o que ela mensura é muito mais fidedigno, sabe, porque ela traz um número muito mais real pra gente” (GF2)	“(...)ela é ótima ela traz números muito reais” (GF2)
		Agilidade na visualização dos dados	“eu acho bacana [ferramenta] eu acho que assim exatamente aquilo né você tem mais visível tudo o que tem na área” (GF1)	“eu concordo com o que ela disse sim também tem aquela parte do alerta né e a gente pode colocar que é um diabético está fazendo uso da medicação (...) é mais fácil pra gente ver, localizar essa visualização ajudou de ajudou muito ajudou bastante“ (GF1)
		Redução no volume de instrumentos utilizados e otimização do tempo	“(...) então eu não preciso pegar todas aquelas fichas para saber quem são os hipertensos e separar por ali, ali é um mapa da minha área” (GF2)	“ (...)o fato de você ter todo o processo de trabalho a mão ali durante a visita que eu já eu faço a visita no ESUS e já lançou na colmeia é muito rápido sabe e economiza tempo” (GF1)
	Usabilidade em atividades de rotina	Caracterização /mapeamento do território e identificação de subpopulação	“(...)a colmeia ajuda bastante mesmo, né, na caracterização do território, ajuda mesmo, né. Como a gente já mencionou, é, ajuda a gente a saber aí, é, tanto da população, né, quantas crianças eu tenho menor de 1 ano menor de 2, quantas crianças, quanta...é, idosos, hipertensos, essas coisas que tem hoje no marcador (...) da pra eu identificar através da colmeia aonde eu tenho minha casa vazia, onde tem a minha casa com pessoas que eu tenho que oferecer cadastro, aonde que tem eu minha casa que é que já recusou o cadastro.” (GF2)	“(...)consegue sim fazer um estudo da área, né, aí essa área tem mais hipertenso, né, essa área tem mais idosos” (GF2)
		Planejamento e monitoramento das visitas domiciliares	“ela também auxilia muito na questão das visitas que a gente faz para saber exatamente quando foi feita para não repetir (...) na colmeia a gente consegue saber certinho que dia que foi feito para não estar fazendo isso de novo tem todo esse controle de revisita também essa parte é muito boa” (GF1)	“diariamente a gente vê nossas visitas a gente tem um controle maior das visitas que nós perdemos e aí da quantidade que já foi feita.” (GF2)
		Registro da produção	“o fechamento a gente usa agora com base de tudo da colmeia (...)eu uso pra saber qual a quantidade de gestante que eu tenho (...) eu acho que no cotidiano a gente usa muito os dados da colmeia, muito, muito, muito. No dia a dia a gente usa muito mesmo, eu não tenho um dia que eu não abro a minha colmeia ou pra fazer visita, ou para fazer a consulta de algum dado, não tem um dia” (GF2)	“No fechamento utilizamos bastante para termos uma base real do nosso território” (GF2)
Avaliação do processo de implementação da ferramenta digital	Aceitabilidade da ferramenta digital	Satisfação, usabilidade e pertinência	“(...)pra mensurar assim indicadores, ela é ótima, eu amei, que na verdade é o nosso cronograma mesmo, só que digitalizado, né, então eu particularmente eu gostei bastante, só tenho a complementar ele, eu não mudaria nada, só aumentar, só melhorar, digamos assim.” (GF2)	“por exemplo a minha questão é sempre essa questão burocrática dos papéis, porque se você tem um tem um aparelho com todo esse potencial para ser explorado e aí você faz parte do trabalho digital a gente fica na unidade tem que lidar com o papel, então a colmeia é muito melhor justamente por causa disso, porque eu tô na casa da paciente eu posso tanto quanto fazer a visita no ESUS e já preencher a colmeia porque está tudo ali na mão.” (GF1)
		Disponibilidade para mudança, adaptação e engajamento do profissional	“foi muito interessante porque como tudo novo gera uma certa preocupação né o ruim é quando foi falado que teria a implantação da colmeia, a gente ficou meio que nossa vem mais trabalho. Como a colega relatou às vezes a gente tem muita demanda né e aí foi uma preocupação muito grande a demanda aumentar, mas quando a gente começou a fazer, pelo contrário será bem positivo é como já foi falado ajuda muito pelo menos de realizar um fechamento. No final do mês é bem interessante” (GF1)	“eu ainda tive dificuldade no primeiro mês né porque realmente não era só colmeia, tinha outras coisas também que a gente precisava correr com o tempo, mas agora esse mês já senti que isso pra mim na relacionada colmeia já temos uma realidade diferente, mais pra ajuda mesmo (...)” (GF1)
Avaliação do processo de implementação da ferramenta digital	Desafios e barreiras para uso na rotina	Restrições do recurso tecnológico	“(...)não faz muito sentido você ter um acesso porque às vezes ele demora bastante dependendo do local que você tá, né, eu pelo menos faço meu território é de vielas então eu tenho uma dificuldade de acesso nesse território, né, e assim, ele demora um pouco as vezes pra abrir né” (GF2)	“(...)eu acho que quando tem muita gente usando o mesmo tempo ele tem um trabalho né isso tem que estar atualizando, aí você fecha a aba e abre de novo.” (GF1)
		Preferência por outros recursos/estratégias	“A facilidade assim da gente ter muito tempo na área é que eu tenho boa parte de tudo isso na cabeça, né. A colmeia é mais para o fechamento, ela não é tão habitual no meu dia a dia.” (GF2)	
		Dificuldade de manejar a ferramenta digital	“(...)vários colegas aqui, que por experiência, né, a gente vê e a gente ajuda, tem bastante dificuldades nas revisitas e colocar a revisitas na colmeia” (GF2)	“Recentemente a gente teve uma ACS nova na nossa equipe e ela ficou bem perdida de ver a colmeia. Ela não entendeu muito (...) até também era muito novo para gente, a gente teve pouco tempo de treinamento, né, pra poder aprender e inserir. (...)treinamento num dia no outro estava de férias. Quando eu voltei foi, fiquei quebrando a cabeça, então não conseguia me explicar então foi um pouquinho difícil.” (GF2)
		Dinâmica da visita domiciliar	“a gente tá com o relatório na mão, a gente tá com o tablet na mão e aí a gente tem que colocar na colmeia, e isso querendo ou não, atrasa um pouco a visita né” (GF2)	“Eu tento fazer e utilizar minha colmeia no momento real da minha visita, as vezes é um pouco complicado em vista de tanta coisa que a gente tem que fazer com a visita, que é fazer visita pelo tablet, além disso, é, fazer o relatório, né, fazer o preenchimento da colmeia, tirar as dúvidas do paciente, passar as orientações pro paciente, então, as vezes a gente começa a eliminar algumas coisas que não é essencial a gente fazer ali pra otimizar mais, aí, o tempo na visita, né (...)” (GF2)
Aperfeiçoamento e oportunidades de melhoria	Revisão em elementos existentes	Adaptação nos marcadores	“E sim, tem muito a melhorar também. (...)porque tem hipertensos e diabéticos somados junto (...) eu acho que poderia também meio que separar isso, sabe.” (GF2)	“(...)talvez colocar algum campo porque tem recusa de ACS e tem a recusa do paciente que não quer que a gente vá, então são os dois tipos de recusa. Eu sei que no campo tem um lá, né, mas acho que também poderia acrescentar alguma coisa sobre isso, porque não tem, né.” (GF2)
		Ajustes no layout	“(...)tem uma coisa que me incomoda tá lá é quando eu entro na colmeia eu acho muito pequenininho o espaço né. O espaço que a gente tem para poder jogar lá e as datas eu acho ruim assim relacionado à quando você vai colocar já que a gente está falando de melhorias né então assim eu acho que porque para mim é a visualização do instrumento não é tão eficiente para mim, às vezes eu coloco lá por exemplo a numeração aí a outra parte da tela some então eu não consigo visualizar tem que esperar um tempo para poder essa parte é voltar para mim conseguir jogar essas numerações nas restantes então pra mim eu acho que tem que ter uma melhoria nesse sentido.” (GF1)	
	Inclusão de novos campos	Inclusão de campo de relatório	“(...)e a pergunta que não quer falar que ia ficar mais maravilhoso ainda se também pudesse ter ou em outra ferramenta um relatório igual que nem era do EPHEALTH.” (GF1)	“99% dos ACS acho que concorda em que se conseguisse incluir o relatório na colmeia seria maravilhoso perfeito.” (GF1)
		Inclusão de campo de observações	“(...)se tivesse um campo para preenchimento de informações que tanto o enfermeiro quanto o médico pudesse estar visualizando né eu acho que seria muito mais efetivo” (GF2)	
		Inclusão de novos marcadores de subpopulação	“(...)eu acho que a gente tem muito além de hipertensos, diabéticos, gestantes, acamados, né, pra colocar que a gente acompanha aí, tem idosos, né, que a gente acompanha, tem mulheres em idade fértil, que são pontos que a gente também precisa contabilizar aí, né, a gente precisa ter o controle” (GF2)	“o somente isso que a colega pontuou aí de todos os marcadores que são que estão nos na nossa meta fossem inclusos também na colmeia (...) então se a gente pudesse colocar todos os marcadores que a gente tinha que a gente acompanhar seria bastante interessante” (GF1)
		Inclusão da identificação nominal das subpopulações	“Seria interessante sinalizar quem são as pessoas hipertensos e diabéticos” (GF2)	“acho que tentar marcar, ter algum marcador que possa, é, que uma outra pessoa possa visualizar quem é essa pessoa” (GF2)
		Inclusão de campo quantificador de famílias e visitas realizadas	“sabe o que eu acho que falta na colmeia? (...)a quantidade de visita realizada naquele dia, entendeu?” (GF2)	“(...)uma coisa também que eu tenho um pouco de dificuldade, que eu fico vendo, é pra totalizar a quantidade do número de família, vamos supor: nesse momento agora eu tenho 213 famílias só pra mim poder ter essa contagem, eu tenho de ficar contando um por um, sabe?” (GF2)

GF1: Grupo focal 1; GF2: Grupo focal 2.

## DISCUSSÃO

Os principais instrumentos utilizados na gestão do território e para acesso aos dados pelas eSF foram planilhas e cadernos pelos ACS, acarretando a dificuldade de gestão dessas informações. Os maiores desafios relatados para acesso aos dados do território foram infraestrutura/rede de internet e a existência de múltiplos SIS para registros das informações, bem como a falta de tempo. Após uso inicial da FAMILY, na perspectiva dos ACS, foram levantados aspectos como integração da ferramenta no cotidiano de trabalho, avaliação do processo de implementação da ferramenta digital, aperfeiçoamento e oportunidades de melhoria são aspectos importantes para potencializar a ferramenta no cotidiano de prática das equipes na APS.

O trabalho do ACS integrado com a equipe multiprofissional na eSF é fundamental para o conhecimento do território a partir do processo de territorialização em saúde. No entanto, a prática cotidiana evidencia fragilidades no seu processo de trabalho e na comunicação com o restante da equipe, agravadas pela existência de sistemas de informação desconexos, conforme demonstram estudos nacionais^2,11^.

A maioria dos ACS referiu dificuldade para atualização das informações do território devido à perda de dados após alimentação dos sistemas. Os achados convergem com resultados de uma pesquisa conduzida no Quênia e Malawi, que investigou a coerência dos dados reportados por ACS em contextos de programas de saúde comunitária em países de renda média e baixa. Constatou-se que somente 15% dos dados coletados pelos ACS foram reportados de maneira consistente a seus supervisores^12^. Essa congruência de resultados aponta para desafios recorrentes que afetam a qualidade dos dados em diferentes contextos: a indisponibilidade de ferramentas adequadas para a coleta e o relato de informações; as deficiências no treinamento e supervisão dos ACS e, a ausência de mecanismos eficazes de controle de qualidade e registros inadequados.

Os participantes do presente estudo relataram que precisavam acessar cerca de seis ferramentas para identificar dados de três subpopulações alvo. Essa tendência de fragmentação persiste no Brasil e é um desafio a ser superado para que a gestão de base populacional seja uma realidade na APS. No período de 2013 a 2018, estavam disponíveis 31 sistemas nacionais de informação em saúde em uso na APS, sendo que, 15 destes não apresentavam nenhuma unificação de interfaces com o PEC/e-SUS APS^2^.

A mitigação dos problemas apontados na fase pré-implantação da ferramenta foi abordada nas Diretrizes da Organização Mundial de Saúde (OMS)^13^ sobre política de saúde e apoio sistêmico para a otimização de programas de ACS. Neste sentido, a FAMILY apresenta-se como uma ferramenta com potencial para contribuir com a organização e otimização da coleta e monitoramento dos dados no território da APS, promovendo maior segurança e confiabilidade destes.

Quando avaliamos a “integração da ferramenta no cotidiano do trabalho”, a ferramenta demonstrou auxiliar na realização do diagnóstico territorial, bem como no monitoramento e organização do processo de trabalho da eSF no território, indo ao encontro dos pressupostos estabelecidos pela PAS, no que tange ao macroprocesso de territorialização e a necessidade de conhecimento da população adscrita, incluindo os aspectos sociais e sanitários, caracterização e registro das subpopulações, alertas e cadastramento desses indivíduos e necessidade de implantação de SIS efetivos^5^.

A adoção de tecnologias móveis no trabalho do ACS tem potencial para contribuir na melhoria do processo de trabalho, desenvolvimento de intervenções mais assertivas, coletas de dados mais completas, de alta qualidade e oportunas. Revisão sistemática da literatura norte americana evidenciou que comparado ao uso de papel, o uso das tecnologias permitiu redução dos erros e perda de dados, além de facilitarem sua revisão e análise em tempo real para tomada de decisões, bem como resposta rápida aos problemas de saúde^14^.

Um outro desafio a ser superado é a incorporação do uso das informações do território na tomada de decisão e organização do processo de trabalho no cotidiano das unidades de APS, ação essencial no contexto da PAS. Apesar de mais da metade dos profissionais informar que possui acesso aos dados, apenas uma pequena parcela destes relatou estruturar as agendas com base nessas informações. Nesta perspectiva, a implantação da FAMILY, que é de fácil manuseio, na rotina de trabalho do ACS, integrada com os demais profissionais do serviço de saúde, potencializa o trabalho em equipe, a comunicação, o reconhecimento de casos que precisam de maior atenção, priorização do cuidado e a gestão de base populacional, constatações demonstradas na análise dos códigos presentes nas falas dos ACS relacionadas à usabilidade, favorecendo a organização de macro e micro processos da APS, por meio da PAS.

Em relação ao processo de implementação da ferramenta digital, embora exista um reconhecimento da sua usabilidade e pertinência, há relatos pelos ACS dos limites no uso da tecnologia, relacionadas ao manuseio e entendimento, dificuldade de acesso à internet em alguns locais, medo de uso do tablet devido ao risco de furtos, bem como lentidão temporária no acesso à ferramenta.

Em um estudo qualitativo realizado com profissionais de nível superior que atuavam em equipes de Saúde da Família do Ceará, apesar das equipes quase não utilizarem tecnologias da informação e comunicação (TIC), eles reconheciam que seu uso oportunizaria maior velocidade no acesso às informações e facilidade na avaliação das intervenções de cuidado prestado. Identificaram também a necessidade de registro e arquivamento adequado das informações, o que pode ser facilitado pelo uso de TIC^15^.

A Agenda 2030 para o Desenvolvimento Sustentável reconhece a necessidade de aumentar o acesso às tecnologias de comunicação e informação. O uso de tecnologias móveis sem fio para a saúde pública ou “mHealth”, é parte integrante do “e-Health”, que se refere ao uso econômico e seguro da informação e comunicação e das tecnologias de apoio à área da saúde. Neste sentido, as tecnologias digitais estão se tornando um importante recurso para a prestação de serviços de saúde^16^.

A implantação de tecnologias de saúde digitais, apesar de ser central para o desenvolvimento dos sistemas de saúde, pode ser difícil de concretizar em países de baixa e média renda. A OMS reconhece ser fundamental o investimento em esforços para superar os principais impedimentos que os países em desenvolvimento enfrentam para se engajarem no uso de novas tecnologias digitais de saúde, como ambiente propício, infraestrutura, educação, capacidade humana e financeira, conectividade com a internet, propriedade de tecnologia. Diante deste quadro, propõe-se a colaboração entre os países membros, com a criação de políticas nacionais pensadas para a realidade de cada país, que direcionem o desenvolvimento das estratégias^17^.

Quanto ao aperfeiçoamento e oportunidades de melhoria da FAMILY, foram destaques os desejos dos ACS de inclusão de um campo para registro do relatório de visita, de forma a reduzir ainda mais o número de instrumentos de trabalho, inclusão da relação nominal das pessoas e inclusão de novos marcadores. Uma vez que as adequações podem ser feitas ao longo do processo de implementação, demonstra-se a aceitabilidade, aplicabilidade e potência da FAMILY como ferramenta de apoio para gestão de base populacional.

Apesar dos resultados satisfatórios e possibilidade de ajuste da ferramenta para diferentes realidades, as limitações deste estudo envolvem o cenário específico em que a pesquisa foi realizada, sendo importante desenvolver novos estudos que analisem os desafios e particularidades da implantação da FAMILY em diferentes regiões. Além disso, destaca-se que a ferramenta não opera em modo offline, o que significa que sua funcionalidade depende de uma conexão 4G constante. Nos casos em que a conexão 4G não está disponível, os ACS realizam os registros em formato físico em papel e, posteriormente, digitam essas informações na ferramenta quando a conectividade for restaurada. Todavia, é uma ferramenta de baixo custo de desenvolvimento e as adaptações podem ser realizadas de acordo com o perfil e necessidade do território.

Conclui-se que, com a implantação da FAMILY, há maior fidedignidade e agilidade na visualização dos dados, redução no volume de instrumentos manuais e otimização do tempo, permitindo um mapeamento visual do território por meio do uso de cores e organização espacial das famílias num mesmo painel e, desta forma, transformando dados em informação de fácil acesso aos ACS e eSF. Ressalta-se, ainda, que a ferramenta não substitui nenhum sistema de informação existente na APS, e sim, oferece funcionalidades adicionais e complementares, como mapeamento de vulnerabilidades, alertas para priorização de atendimentos, acesso facilitado a relatórios e indicadores de desempenho, além de uma interface intuitiva para visualização de dados em tempo real.

Espera-se que a FAMILY, disponibilizada no material suplementar, possa ser incorporada por outros municípios de forma a contribuir com a operacionalização dos macroprocessos básicos da APS e aprimoramento da gestão de base populacional conforme proposta da PAS.
